# Effects of pomegranate seed oil on oxidant/antioxidant balance in heart and kidney homogenates and mitochondria of diabetic rats and high glucose-treated H9c2 cell line

**Published:** 2017

**Authors:** Hamid Mollazadeh, Mohammad Taher Boroushaki, Mohammad Soukhtanloo, Amir Reza Afshari, Mohammad Mahdi Vahedi

**Affiliations:** 1 *Department of physiology and pharmacology, School of Medicine, North Khorasan University of Medical Sciences, Bojnurd, Iran*; 2 *Department of Pharmacology, Faculty of Medicine, Mashhad University of Medical Sciences, Mashhad, Iran*; 3 *Pharmacological Research Center of Medicinal Plants, Mashhad University of Medical Sciences, Mashhad, Iran*; 4 *Department of Biochemistry, School of Medicine, Mashhad University of Medical Sciences, Mashhad, Iran*; 5 *Health promotion Research Center, Zahedan University of Medical Science, Zahedan, Iran*

**Keywords:** Pomegranate Seed Oil, Diabetes mellitus, Oxidant, Antioxidant, Oxidative stress

## Abstract

**Objective:**

Oxidative stress is a major cause of diabetes complications. The present study aimed to investigate the beneficial effects of Pomegranate Seed Oil (PSO) on diabetes-induced changes in oxidant/antioxidant balance of the kidney, heart and mitochondria from rats and H9c2 cell line.

**Materials and Methods:**

In these *in vivo* and *in vitro* studies, male rats were divided into four groups (twelve each): group 1 served as control, group 2-4 received a single dose of streptozotocin (60 mg/kg, i.p), groups 3 and 4 received PSO (0.36 and 0.72 mg/kg/daily, gavage), respectively. After three weeks, six rats of each group and one week later the remaining animals were anaesthetized and the hearts and kidneys were removed and homogenized. Mitochondrial fractions were separated and enzyme activities were measured in each sample. H9c2 cells were pretreated with high levels of glucose (35 mM), and then, incubated with PSO. Finally, cell viability test, reactive oxygen species production and lipid peroxidation were evaluated.

**Results:**

Significant reduction in enzymes activity (Superoxide dismutase, Glutathione S-transferase and Paraoxonase 1), compensatory elevation in Glutathione Reductase, Glutathione Peroxidase and Catalase activity followed by reduction after one week and significant elevation in Oxidative Stress Index (OSI) were observed in diabetic group. PSO treatment resulted in a significant increase in enzymes activity and decreased OSI values compared to diabetic group in both tissue and mitochondrial fractions. PSO remarkably decreased glucose-induced toxicity, ROS level and lipid peroxidation in H9c2 cells.

**Conclusion:**

Results suggested that PSO has a protective effect against diabetes-induced alterations in oxidant/antioxidant balance in tissues, mitochondrial and H9c2 cell line.

## Introduction

Diabetes mellitus (DM) is a chronic and life-threatening illness characterized by high amount of glucose and high levels of glycated hemoglobin (HbA1C) in serum which might be due to adverse living conditions, genetic predisposition factors and dietary habits (TA 2014 ▶). Industrialized and developing nations have higher incidence and prevalence of DM. DM as a chronic disease affects all organs and systems of the body and retinopathy, neuropathy, nephropathy and cardiovascular diseases are the main causes of morbidity and mortality in DM due to its micro and macrovascular complications. One of the most important factors responsible for cell disruption in DM is oxidative stress (OxS) caused by chronic hyperglycemia (Forbes and Cooper 2013 ▶; Mollazadeh, Sadeghnia, Hoseini, Farzadnia and Boroushaki 2016 ▶). 

OxS is defined as an imbalance between free radical production and antioxidant capacity in the body. In the process of oxidation-reduction, detached electrons are formed which could induce detrimental effects on human organs. OxS is increased in parallel with the development of DM and interferes with normal cellular signaling and functions (Baynes and Thorpe 1999 ▶). Glucose auto-oxidation, non-enzymatic glycosylation, increased production of free fatty acids, activation of sorbitol pathway, activation of pentose phosphate pathway, increased production of inflammatory cytokines and activation of hexosamine pathway alongside with reduced antioxidant defense are the main mechanisms of increasing OxS. Amelioration of OxS represents an effective way to reduce the adverse consequences of reactive oxygen species (ROS). It has been suggested that stronger antioxidant system may decrease OxS and prevent DM progression and its complications (Johansen, Harris, Rychly and Ergul 2005 ▶; Rains and Jain 2011 ▶; Valko, Leibfritz, Moncol, Cronin, Mazur and Telser 2007 ▶).

Pomegranate (*Punica granatum *L.), from family Punicaceae, has been traditionally used as a medicinal and Ayurvedic fruit for its antioxidant, anti-proliferative, antimicrobial, anti-inflammatory, cardiovascular protective, anti-diabetic and anti-obesity properties. Pomegranate seed oil (PSO) which approximately comprises 12–20% of total seed weight is a rich source of conjugated octadecatrienoic fatty acids including conjugated linolenic acids (CLnAs). PSO has been reported to have beneficial therapeutic effects including body fat-reducing and lipid metabolism-normalizing and antitumor properties. Also, it can reduce body weight and leptin and insulin levels while it increases glucose tolerance and peripheral insulin sensitivity. Increasing carbohydrate oxidative capacity, inhibiting progression of type-2 diabetes, and normalizing lipid profile are other therapeutic features of PSO. Beneficial health effects of PSO, especially its antioxidant and anti-inflammatory activities are due to the presence of high level of CLnAs (Adams, Seeram, Aggarwal, Takada, Sand and Heber 2006 ▶; Boroushaki, Mollazadeh and Afshari 2016 ▶; Boroushaki, Mollazadeh, Rajabian, Dolati, Hoseini, Paseban and Farzadnia 2014 ▶; Goertz and Ahmad 2015 ▶; Vroegrijk, van Diepen, van den Berg, Westbroek, Keizer, Gambelli, Hontecillas, Bassaganya-Riera, Zondag and Romijn 2011 ▶).

Nowadays, the role of natural products as alternative sources of medicinal compounds is highly appealing for researchers. Use of herbal antioxidant supplements has been increased due to their safety, acceptability and easy to earn; therefore, investigators try to develop therapeutic agents with antioxidant effects (Mollazadeh and Hosseinzadeh 2014 ▶). Thus, this study was designed to evaluate the effects of DM and PSO, as a rich source of unsaturated fatty acids on antioxidant-oxidant status in heart and kidney tissues as well as their mitochondrial fractions, in rats with hyperglycemia induced by streptozotocin (STZ). Moreover, we decided to evaluate the effects of high glucose medium on viability of H9c2 cell line and the protective effects of PSO against glucose-induced toxicity based on its OxS inhibition mechanism. 

## Materials and Methods

**Animals**

Forty-eight adult male wistar rats (8 weeks old and weighing 235–275 g) which were purchased from Animal House, School of Medicine, Mashhad University of Medical Sciences, Mashhad, Iran , were used in this study. These animals were housed in a clean rodent room under a 12-hr: 12-hr light-dark cycle, with *ad libitum* access to food and water at a temperature of 24±1^°^C. The rats were fed with standard diet composing of fat (2-3%), starch (55 %), protein (20-21%), crude fiber (6%), NaCl (5%), trace elements and amino acids. All animal procedures were approved by Ethics Committee of Mashhad University of Medical Sciences and were done in compliance with National Laws and with National Institutes of Health guidelines for the use and care of laboratory animals.

***In vivo*** **procedure** **s**

**Experimental design and induction of diabetes using STZ**

After acclimatization, animals were randomly divided into four groups (12 each) and individually put in metabolic cages. Group 1 served as control and orally received saline (1 ml/kg/day). Groups 2, 3 and 4 were injected with a single dose of STZ (65mg/kg body weight, i. p., dissolved in 0.1M citrate buffer, pH: 4.5), three days before PSO administration. PSO (d=0.81 g/mL was diluted in DMSO at 25 ^0 ^C and it was a kind gift from Urom Narin Co, Uromeya, Iran). The induction of DM in rats was confirmed by blood glucose evaluation after 48 hr. After overnight fasting, animals with blood glucose higher than 250 mg/dl (blood samples were obtained from the tail vein of rats and detected by a strip-operated blood glucose meter (Accu-Check Commpact Plus, Roche Diagnostics, Germany), were considered as diabetic and selected for studies. Group 3 and 4 were treated daily with oral PSO 0.4 and 0.8 ml/kg, respectively. The doses of PSO and STZ were selected on the basis of previous studies (Boroushaki, Mollazadeh, Rajabian, Dolati, Hoseini, Paseban and Farzadnia 2014 ▶; Mollazadeh, Sadeghnia, Hoseini, Farzadnia and Boroushaki 2016 ▶). All procedures were performed between 10:00 and 12:00 AM. After overnight fasting, animals were immediately anesthetized by injection of ketamine (60 mg/kg) and xylazine (10 mg/kg). The kidneys and heart were quickly excised for enzymatic assay studies and stored at -70^°^C. One week later, the remaining animals in each group, underwent the previous process. 

**Homogenization protocol**

One kidney and heart were scissor-minced, washed with cold isotonic normal saline (0.9%, 4°C) and homogenized in ice shower containing 4ml of 0.2M phosphate buffer with pH:7.4 (Heidolph hemogenizer, Germany). Homogenates were centrifuged at 3,000 g for 15 min at 4^°^C to remove tissue remnants. Homogenates were restored at -70^°^C until further analysis.

**Mitochondria extraction**

Mitochondria Extraction was performed according to a previously reported method (Max, Garbus and Wehman 1972 ▶). In this method, the heart tissue and 1 g of kidney were homogenized in 5mL of 0.25M cold sucrose solution. Homogenates were centrifuged at 800 g for 15 minutes at 4°C and then supernatant was re-centrifuged at 20,000 g for 10 minutes. After that, supernatant was removed and the leniently inflated layer on top of the mitochondrial pellet was raised with the mild addition of sucrose solution. Pellet was later prepared for analysis by suspension with 1.2 mL of 0.02 M phosphate buffer at pH: 7.4.

**Activity analysis**

All markers analysis was performed in tissue homogenates and mitochondria extraction separately as mentioned below:

***TAS, TOS and OSI assay***

Total Antioxidant Status (TAS) and Total Oxidant Status (TOS) levels were measured using the commercial kit manner (Rel Assay Diagnostic, Turkey) with spectrophotometer (Cecil: CE-9500) auto-analyzer. Oxidative Stress Index (OSI) was calculated according to the following formula: OSI: TOS/TAS×100.

***Catalase activity assay***

Catalase (CAT) activity was determined according to the method of Abei (Aebi 1984 ▶). Homogenates were kept at 4 ^◦^C in 50 mM potassium phosphate buffer (pH: 7.4) and centrifuged at 5000 rpm for 10 min. Ethanol equivalent to 0.01 ml/mL of supernatant was included and brooded for 30 min in ice. Triton 100× was added to a last centralization of 1%. Supernatant was added to a cuvette containing 1.95 ml of 50 mM phosphate buffer. At that point 1.0 ml of 30 mM hydrogen peroxide (H_2_O_2_) was included and rate of deterioration of H_2_O_2_ was measured at 240 nm. Catalase activity was calculated according to the following formula as unit/mg protein: 

 Activity = [ΔA × 0.73] / 0.00348

***Superoxide dismutase activity assay***

Superoxide dismutase (SOD) activity was measured by the calorimetric method (Madesh method) based on the inhibition of superoxide anion production due to auto-oxidation of pyrogallol. Reaction between 4, 5-dimethylthiazole-2-yl, 2, 5-diphenyl tetrazolium (MTT) and superoxide anion was diminished by the enzyme activity (Madesh and Balasubramanian 1998 ▶). SOD activity was expressed as U/mg protein.

***Glutathione S-transferase activity assay***

Glutathione S-transferase (GST) activity was measured spectrophotometrically at 340 nm according to the method described by Habig et al. In this method, the reaction between 2, 4-dinitrochlorobenzene (CDNB) and glutathione in the presence of GST, leads to the enhancement of absorbance. GST activity was expressed as U/mg protein (Habig, Pabst and Jakoby 1974 ▶).

***Glutathione reductase activity assay***

Glutathione Reductase (GR) activity was measured according to the method defined by Paglia (Paglia and Valentine 1967 ▶), based on the amount of oxidized glutathione (GSSG) oxidation in the presence of NADPH into reduced glutathione (GSH) and the reduction in light absorption at 340 nm. GR activity was expressed as U/mg protein.

***Glutathione peroxidase activity assay***

Glutathione Peroxidase (GPx) activity was measured spectrophotometrically at 340 nm according to the commercial kit's instructions (GPx Assay Kit, Cayman, USA). GPx activity was expressed as U/mg protein.

***Paraoxonase 1 activity assay***

Paraoxonase 1 (PON1) activity was measured spectrophotometrically at 412 nm according to the method described by Beltowsli et al (Beltowski, Wójcicka and Marciniak 2002 ▶). Para-nitrophenol production from paraoxon by PON1, leads to the reduction in light absorption. PON1 activity was expressed as U/l. 

***In vitro*** **studies**

***Cell line and substances***

H9c2 cell line was purchased from Pasteur Institute (Tehran, Iran). MTT, Dichloro-dihydro-fluorescein diacetate (DCFH-DA) and Dulbecco’s phosphate-buffered saline (PBS) were purchased from Sigma (St Louis, MO, USA). Glucose-high Dulbecco’s modified Eagle’s medium (DMEM), fetal bovine serum (FBS), penicillin and streptomycin were purchased from Gibco (Grand Island, NY). Dimethyl sulfoxide (DMSO), 2-thiobarbituric acid (TBA) and trichloroacetic acid (TCA) were purchased from Merck. Sodium citrate and Triton X-100 were purchased from Sigma (St. Louis, MO, USA). 

***Cell culture***

H9c2 cells were cultured in high glucose DMEM (4.5 g/L) supplemented with 10% FBS and 100 U/mL of penicillin/streptomycin. All cells were maintained in a 90% humidified atmosphere containing 5% CO_2_ at 37^°^C.

***Cell proliferation (MTT) assay***

H9c2 cells (5000/well) were seeded out in 96-well culture plates, and then the cells were treated with PSO (12-800 𝜇g/ml). After this procedure, non-toxic concentrations of PSO were used for treatment of cells. For this purpose, cells were pretreated with glucose solution (35 mM) for 24 hr and then incubated for 24 or 48 hr with non-toxic concentrations of PSO. MTT solution in phosphate-buffered saline (5 mg/ml) was added to each well at the final concentration of 0.05%. After 3 hr, the absorbance was measured at 570 and 620 nm (background) using a Stat FAX303 plate reader. All treatments were carried out in triplicate.

***Measurement of reactive oxygen species***

Intracellular reactive oxygen species (ROS) production was measured in both PSO-treated and control cells using DCFH-DA. Briefly 2×10^5^ cells/well were exposed to various concentrations of PSO for different incubation times (24 and 48 hr). After incubation, cells were washed once with PBS. Treated and control cells were re-suspended in 0.5 ml PBS containing 10 μM DCFH-DA at 37 °C for 30 min and then incubated with glucose (as the inducer of ROS production) at 37 ^°^C for 24 hr. ROS production was exposed by an spectrophotometer. Then, the fluorescence intensity was detected at excitation/emission of 485/530 nm. All treatments were carried out in triplicate (Moongkarndi, Kosem, Kaslungka, Luanratana, Pongpan and Neungton 2004 ▶).

***Lipid peroxidation assay***

The level of lipid peroxidation was estimated by measuring MDA which is the end-product of lipid peroxidation. Cells were seeded in 96-well culture plates. After 24 hr, the cells were pretreated (for 24 hr) with PSO (12-200 μg/ml) and then incubated with glucose solution (35 mM) for 3 hr. After that, the cells were scraped and centrifuged for 30 min. Then, 400 μl of TCA (15%) and 800 μl of TBA (0.7%) were added to 500 μl of cell samples. The mixture was vortexed and then, heated for 40 min in a boiling water bath. Then, 200 μl of the sample was transferred to a 96-well plate and the fluorescence intensity was read at excitation/emission of 480/530 nm. All treatments were carried out in triplicate. MDA contents were measured by following formula and showed by percent of MDA production in controls: 

MDA (mol^-1^ cm^-1^) = absorbance / 1.56 × 10^5^

**Statistical analysis**

Statistical analysis was performed using two-way analysis of variance (ANOVA) followed by Bonferroni *post-hoc* test for multiple comparisons (GraphPad Prism, Version 6.01) in *in vivo* experiments and one-way ANOVA followed by Tukey *post-hoc* test was used for multiple comparisons for data analysis in *in vitro* experiments. Data were expressed as mean±SEM and the p-values less than 0.05 were considered to be statistically significant.

## Results

**TAS, TOS and OSI**

TAS, TOS and OSI levels in heart and kidney homogenates are shown in Tables 1 and 2, respectively. Induction of diabetes resulted in a non-significant elevation in TOS in both tissue homogenates. PSO-treated group resulted in a non-significant reduction in TOS value compared with diabetic group and significant reduction compared to control group (p<0.05). An increase in TAS values after the 3^rd^ week, was seen in diabetic group compared with control group, but TAS values decreased after the 4^th^ week more than that of the 3^rd^ week (p>0.05). PSO-treated group resulted in a significant elevation in TAS in both tissue homogenates and this elevation was significant in heart after 4 weeks (p<0.05).

**Table 1:** Effect of streptozotocin and pomegranate seed oil on Total Antioxidant Status (TAS), Total Oxidant Status (TOS) and Oxidative Stress Index (OSI) in kidney homogenates

*Groups*	*Kidney*		
	TAS (mmol trolox Equiv/L)	TOS (μmol H2O2 Equiv/L)	OSI(%)
**Con (3w)**	0.41 ± 0.16	7.15 ± 1.26	17.43 ± 2.15
**Con (4w)**	0.42 ± 0.19	6.95 ± 2.21	16.54 ± 3.16
**STZ Group (3w)**	0.46 ± 0.25	8.15 ± 1.26	17.71 ± 2.63
**STZ Group (4w)**	0.40 ± 0.35	7.72 ± 2.26	19.30 ± 3.55
**PSO (0.4 ml/kg, 3w)**	0.48 ± 0.21 e	6.91 ± 1.24	14.39 ± 2.12 e
**PSO (0.4 ml/kg, 4w)**	0.48 ± 0.61 e	6.95 ± 2.11	14.24 ± 1.57 e
**PSO (0.8 ml/kg, 3w)**	0.51 ± 0.32 e	7.11 ± 2.34	13.94 ± 1.47 e
**PSO (0.8 ml/kg, 4w)**	0.51 ± 0.21 e	6.94 ± 1.28	13.60 ± 3.26 a, e

Data were shown as mean ± SEM.

a p<0.05 as compared with STZ-treated group and

e p<0.01 as compared to control group.

**Table 2:** Effect of streptozotocin and pomegranate seed oil on Total Antioxidant Status (TAS), Total Oxidant Status (TOS) and Oxidative Stress Index (OSI) in heart homogenates

*Groups*	*Heart*		
	TAS (mmol trolox Equiv/L)	TOS (μmol H2O2 Equiv/L)	OSI (%)
**Con (3w)**	0.15 ± 0.26 a	2.15 ± 0.91	14.33 ± 0.95
**Con (4w)**	0.18 ± 0.15	2.21 ± 1.02	12.27 ± 3.15
**STZ Group (3w)**	0.21 ± 0.10	2.89 ± 0.75	13.76 ± 3.21
**STZ Group (4w)**	0.20 ± 0.14	2.92 ± 0.86	14.60 ± 2.95
**PSO (0.4 ml/kg, 3w)**	0.21 ± 0.05	1.92 ± 0.61 e	9.14 ± 2.12 b, e
**PSO (0.4 ml/kg, 4w)**	0.22 ± 0.11	1.66 ± 0.25 e	7.54 ± 1.24 b, e
**PSO (0.8 ml/kg, 3w)**	0.25 ± 0.14	1.48 ± 0.31 e	5.92 ± 1.15 b, f
**PSO (0.8 ml/kg, 4w)**	0.26± 0.09 a	1.62 ± 0.24 e	6.23 ± 2.18 b, f

Data were shown as mean±SEM.

a p<0.05 and

b p<0.01 as compared to STZ-treated group and

e p<0.01 and

f p<0.001 as compared to control group.

A non-significant elevation in OSI value was seen in diabetic group compared with control group in both tissue homogenates but a significant reduction in OSI was seen in PSO-treated groups in both tissue homogenates. This reduction was more marked in the heart than kidney (p<0.01 and p<0.05, respectively).

TAS, TOS and OSI levels in heart and kidney mitochondria are shown in Tables 3 and 4, respectively. More marked increases in TOS and OSI values and decreases in TAS value were observed in heart mitochondria than in kidney mitochondria extract in diabetic group compared with control group (p<0.05). Similarly, PSO-treated groups showed a significant elevation in TAS, and significant reductions in TOS and OSI values in heart mitochondria compared to diabetic group (p<0.05 and p<0.01).

**Table 3:** Effect of streptozotocin and pomegranate seed oil on Total Antioxidant Status (TAS), Total Oxidant Status (TOS) and Oxidative Stress Index (OSI) in kidney mitochondria

*Groups*	*Kidney*		
	TAS (mmol trolox Equiv/L)	TOS (μmol H2O2 Equiv/L)	OSI (%)
**Con (3w)**	1.46 ± 0.41	2.39 ± 0.16	1.63 ± 0.28
**Con (4w)**	1.44 ± 0.52	2.44 ± 0.21	1.69 ± 0.18
**STZ Group (3w)**	1.41 ± 0.27	2.34 ± 0.58	1.65 ± 0.67
**STZ Group (4w)**	1.42 ± 0.62	2.43 ± 0.64	1.71 ± 0.34
**PSO (0.4 ml/kg, 3w)**	1.51 ± 0.74	2.51 ± 0.51	1.66 ± 0.24
**PSO (0.4 ml/kg, 4w)**	1.54± 0.68 a	2.53 ± 0.46	1.64 ± 0.19
**PSO (0.8 ml/kg, 3w)**	1.51 ± 0.41	2.58 ± 0.66	1.70 ± 0.09
**PSO (0.8 ml/kg, 4w)**	1.55± 0.51 a	2.52 ± 0.58	1.62 ± 0.1

Data were shown as mean±SEM.

a p<0.05 as compared to STZ-treated group.

**Table 4:** Effect of streptozotocin and pomegranate seed oil on Total Antioxidant Status (TAS), Total Oxidant Status (TOS) and Oxidative Stress Index (OSI) in heart mitochondria

*Groups*	*Heart*		
	TAS (mmol trolox Equiv/L)	TOS (μmol H2O2 Equiv/L)	OSI (%)
**Con (3w)**	0.81 ± 0.29 a	1.29± 0.57 a	1.59 ± 0.21 a
**Con (4w)**	0.85 ± 0.14 a	1.31 ±0.42 a	1.54 ± 0.38 a
**STZ Group (3w)**	0.48 ± 0.24	1.85 ± 0.32	3.85 ± 0.41
**STZ Group (4w)**	0.48 ± 0.12	1.92 ± 0.19	4 ± 0.35
**PSO (0.4 ml/kg, 3w)**	0.51 ± 0.09 d	1.76 ± 0.29	3.45 ± 0.42 e
**PSO (0.4 ml/kg, 4w)**	0.52 ± 0.15 d	1.78 ± 0.36	3.42 ± 0.31 e
**PSO (0.8 ml/kg, 3w)**	0.55 ± 0.25 d	1.69 ± 0.25 a	3.07 ± 0.44 a , e
**PSO (0.8 ml/kg, 4w)**	0.56 ± 0.18 d	1.59 ± 0.31 a	2.82 ± 0.29 a ,e

Data were shown as mean±SEM.

a p<0.05 as compared to STZ-treated group and

d p<0.05 and

e p<0.01 as compared to control group.

**Catalase activity**

CAT activity in heart homogenates was significantly elevated in diabetic group compared to control group (p<0.01). Similarly, this elevation was seen in kidney homogenates but it was not significant. PSO administration resulted in a non-significant elevation of CAT activity compared with control and diabetic groups in both tissues (but it was significant in the kidney after the 4^th^ week compared to control and diabetic groups, p<0.01). 

**Figure 1:**
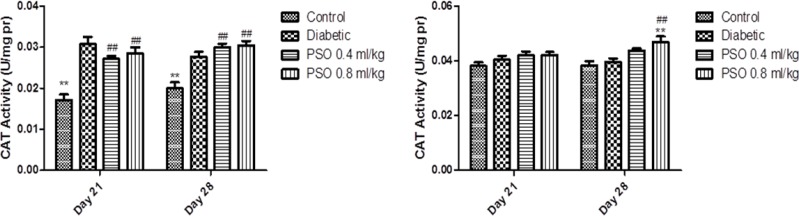
Effect of the pomegranate seed oil treatment on tissue CAT activity in heart (left) and kidney (right) homogenates in a model of STZ-induced diabetes. Values are expressed as mean ± SEM. ** p<0.01 as compared to STZ-treated group and ## p<0.01 as compared to control group

**Figure 2:**
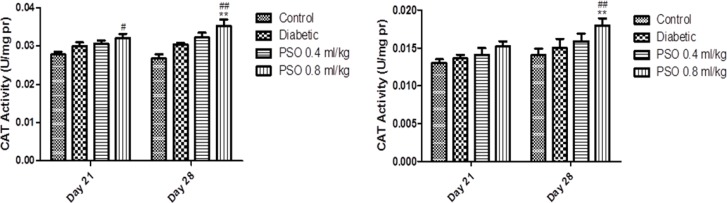
Effect of the pomegranate seed oil treatment on CAT activity in heart (left) and kidney (right) mitochondria in a model of STZ-induced diabetes. Values are expressed as mean ± SEM. ** p<0.01 as compared to STZ-treated group and # p<0.05 and ## p< 0.01 as compared to control group

CAT activity in mitochondria extract was elevated in diabetic group compared with control group in both extracts. PSO resulted in a non-significant elevation in CAT activity compared with control and diabetic groups after 3 weeks, but this elevation became significant after 4 weeks of treatment in both mitochondrial extracts compared to control and diabetic groups (p<0.01, after 4 weeks). Data are shown in Figures 1 and 2, respectively.

**Superoxide dismutase activity**

**Figure 3:**
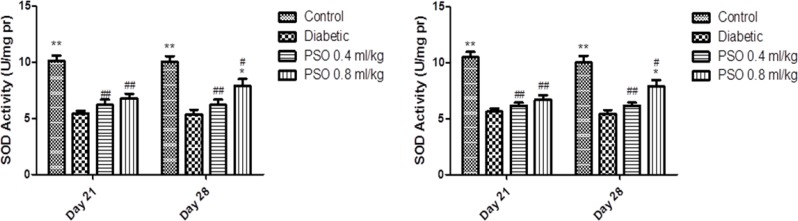
Effect of the pomegranate seed oil treatment on tissue SOD activity in heart (left) and kidney (right) homogenates in a model of STZ-induced diabetes. Values are expressed as mean ± SEM. * p<0.05 and ** p< 0.01 as compared to STZ-treated group and # p<0.05and ## p< 0.01 as compared to control group

**Figure 4:**
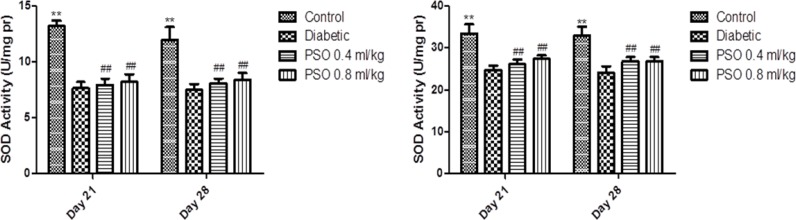
Effect of the pomegranate seed oil treatment on SOD activity in heart (left) and kidney (right) mitochondria in a model of STZ-induced diabetes. Values are expressed as mean ± SEM. ** p<0.01 as compared to STZ-treated group and ## p< 0.01 as compared to control group

SOD activity in both tissue homogenates was decreased significantly in STZ-treated group compared to control group (p<0.01, after 3 and 4 weeks). PSO elevated enzyme activity in both tissues but this elevation was significant in group 4 after 4 weeks (p<0.05). Similarly, SOD activity in both mitochondrial extracts was significantly decreased in STZ-treated group compared to control group (p<0.01, after 3 and 4 weeks). PSO treatment resulted in a non-significant elevation in enzyme activity compared with STZ-treated group. Data are shown in Figures 3 and 4, respectively.

**Glutathione S-transferase activity**

GST activity was significantly reduced after induction of diabetes in both tissue homogenates (p<0.01). PSO elevated enzyme activity in both tissues in a dose and time-dependent manner. This elevation was more marked in heart than kidney homogenates and PSO restored GST activity to near the control group level.

**Figure 5:**
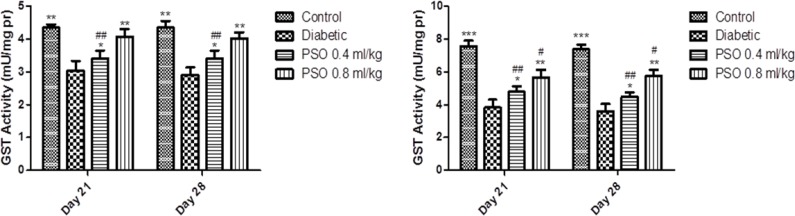
Effect of the pomegranate seed oil treatment on tissue GST activity in heart (left) and kidney (right) homogenates in a model of STZ-induced diabetes. Values are expressed as mean ± SEM. * p<0.05, ** p<0.01 and *** P<0.001 as compared to STZ-treated group and # p<0.05 and ## p<0.01 as compared to control group

**Figure 6:**
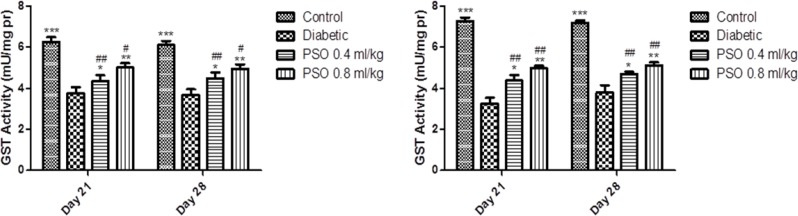
Effect of the pomegranate seed oil treatment on GST activity in heart (left) and kidney (right) mitochondria in a model of STZ-induced diabetes. Values are expressed as mean ± SEM. * p<0.05, ** p<0.01 and *** P<0.001 as compared to STZ-treated group and # p<0.05 and ## p<0.01 as compared to control group

STZ-treated group showed a significant reduction in GST activity compared to control group in mitochondrial extraction (p<0.01). Treatment with PSO significantly increased GST activity (p<0.05 after 3 weeks and p<0.01 after 4 weeks). Data are shown in Figures 5 and 6, respectively.

**Glutathione reductase and glutathione peroxidase activity**

The tissue GR and GPx activity are reported in Table 5. There was a significant increase in GR activity in both tissue homogenates at two time points of study in STZ-treated groups compared to control group (p<0.05) and a significant elevation in PSO-treated groups compared to STZ-treated groups in heart (p<0.05 in group 4 after two times of study). But it was not significant in the kidney. GR activity in PSO-treated groups was significantly increased compared with control group (p<0.05 in group 3, p<0.01 in group 4 in kidney and p<0.05 in two groups of study in heart). 

GPx activity was increased in the kidney (p<0.01) and decreased in the heart (p<0.05) in STZ-treated group compared with control group. PSO caused a non-significant elevation in GPx activity in both tissue homogenates at two time points of study but these values in the kidney were significant compared with control group at two time points of study (p<0.01).

The mitochondrial GR and GPx activity are reported in Table 6. There was a significant increase in both enzymes activity in both extracts at two time points of the study in STZ- treated groups compared to control group (p<0.05 for GPx in the heart and p<0.01 for GR in both extracts and GPx in kidney mitochondrial extracts). Treatment with PSO significantly increased GR and GPx activity in the heart at two time points of the study (p<0.05) but the other elevation in enzymes activity in the kidney and heart in two group was not significant. Elevation in enzymes activity in both extracts in PSO-treatment group compared to control group was significant at the same time points of the study (p<0.01).

**Table 5:** Effect of the pomegranate seed oil treatment on GR and GPx activity in kidney and heart homogenates in a model of STZ-induced diabetes

*Groups*	*Kidney*		*Heart*	
	GR (U/mgPr)	GPx (U/mgPr)	GR (U/mgPr)	GPx (U/mgPr)
**Con (3w)**	6.21 ± 1.7 a	50.12 ± 7.2 b	2.11 ± 0.8 a	70.32 ± 8.9 a
**Con (4w)**	6.52 ± 1.6 a	55.32 ± 6.1 b	2.07 ± 0.6 a	74.87 ±8.2 a
**STZ Group (3w)**	9.13 ± 2.1	90.12 ± 9.5	3.01 ± 0.5	58.25 ± 7.8
**STZ Group (4w)**	8.31 ± 1.9	88.45 ± 8.4	2.78 ± 0.4	55.91 ± 6.8
**PSO (0.4 ml/kg, 3w)**	11.27 ± 2.3 d	95.98 ± 9.9 e	3.11 ± 0.8 d	63.58 ± 8.9
**PSO (0.4 ml/kg, 4w)**	11.15 ± 2.1 d	97.54 ± 9.1 e	3.09 ± 0.9 d	62.44 ± 9.8
**PSO (0.8 ml/kg, 3w)**	12.22 ± 2.5 e	99.25 ± 8.6 e	4.21 ± 0.9 a, d	65.66± 8.1
**PSO (0.8 ml/kg, 4w)**	13.11 ± 2.7 e	105.24 ± 10.1 e	4.28 ± 0.7 a, d	65.75 ± 8.2

Data were shown as mean±SEM.

a p<0.05 and

b p<0.01 as compared to STZ-treated group and

d p<0.05 and

e p<0.01 as compared to control group.

**Table 6:** Effect of the pomegranate seed oil treatment on GR and GPx activity in kidney and heart mitochondria in a model of STZ-induced diabetes

*Groups*	*Kidney*		*Heart*	
	GR (U/mgPr)	GPx (U/mgPr)	GR (U/mgPr)	GPx (U/mgPr)
**Con (3w)**	0.16± 0.02 b	0.25 ± 0.04 b	0.11± 0.02 b	0.17 ± 0.03 a
**Con (4w)**	0.16± 0.02 b	0.27 ± 0.03 b	0.12± 0.02 b	0.16 ± 0.03 a
**STZ Group (3w)**	0.30 ± 0.04	0.39 ± 0.04	0.25 ± 0.06	0.25 ± 0.03
**STZ Group (4w)**	0.28 ± 0.04	0.37 ± 0.04	0.24 ± 0.06	0.24 ± 0.02
**PSO (0.4 ml/kg, 3w)**	0.35 ± 0.05 e	0.42 ± 0.04 e	0.29 ± 0.06^e^	0.29 ± 0.03 e
**PSO (0.4 ml/kg, 4w)**	0.35 ± 0.04 e	0.42 ± 0.04 e	0.29 ± 0.06^e^	0.31 ± 0.05 e
**PSO (0.8 ml/kg, 3w)**	0.39 ± 0.05 e	0.44 ± 0.05 e	0.35 ± 0.07 a, e	0.35±0.05 a ,e
**PSO (0.8 ml/kg, 4w)**	0.39 ± 0.05 e	0.45 ± 0.04 e	0.36 ± 0.06 a, e	0.35±0.03 a, e

Data were shown as mean±SEM.

a p<0.05 and

b p<0.01 as compared to STZ-treated group and

e p<0.01 as compared to control group.

**Paraoxonase1 activity**

**Figure 7:**
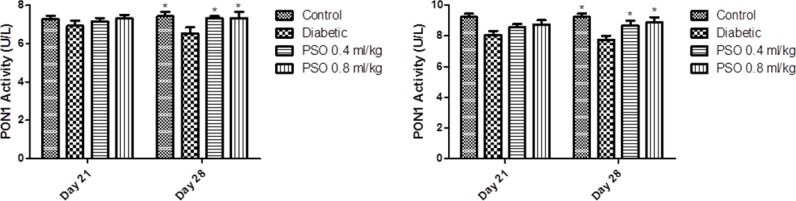
Effect of the pomegranate seed oil treatment on tissue PON1 activity in heart (left) and kidney (right) homogenates in a model of STZ-induced diabetes. Values are expressed as mean ± SEM. * p<0.05 as compared to STZ-treated group

**Figure 8:**
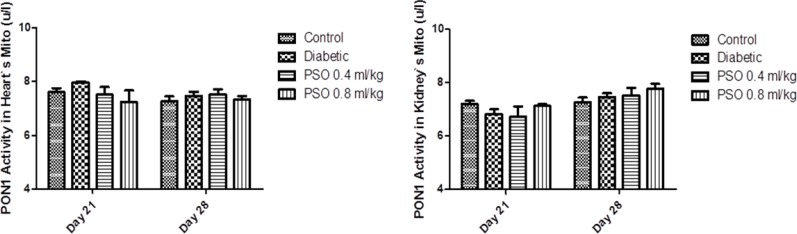
Effect of the pomegranate seed oil treatment on PON1 activity in heart (left) and kidney (right) mitochondria in a model of STZ-induced diabetes. Values are expressed as mean ± SEM

PON1 activity in STZ-treated group was significantly decreased compared to control group after 4 weeks of the study (p<0.05) but its reduction after 3 weeks was not significant. PSO caused significant elevation in enzyme activity after 4 weeks in two tissue homogenates from two treated groups (p<0.05). PON1 activity in mitochondrial extracts did not show any significant changes and increases in its activity in the kidney from two treated groups were not significant. Complementary data was shown in Figures 7 and 8.

**Effect of PSO on cell viability**

**Figure 9:**
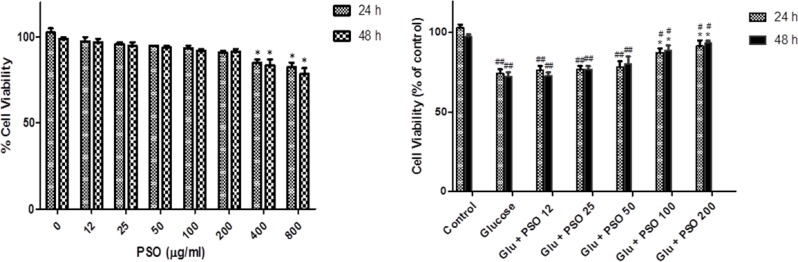
Effect of PSO alone on cell viability in H9c2 (left). The cells were treated (for 24 hr) with different concentrations of PSO. Effect of PSO on glucose-induced cytotoxicity in H9c2 cells (right). The cells were pretreated (for 24 hr) with glucose (35 mM) before to exposure (for 24 and 48 hr) to non-toxic concentration of PSO. Data are expressed as mean ± SEM of three separate experiments. * p<0.05 as compared to glucose-treated group and # p<0.05 and ## p<0.01 as compared to control group

Incubation with PSO for 24 and 48 hr significantly decreased the viability of cells at the concentrations of 400 and 800 g/ml (p<0.05 compared to control). Other concentrations did not reduce cell viability (Figure 9, left).

**Effect of PSO on cell viability against high glucose**

Incubation with high glucose solution significantly decreased cell viability to 75.5 ± 2.5% and 73.5 ± 3.5% of that of control after 24 and 48 hr, respectively (p<0.01). Treatment with PSO significantly increased cell viability compared to control at the doses of 100 and 200 g/ml after 24 and 48 hr (p<0.05) (Figure 9, right).

**Effect of PSO on ROS content**

**Figure 10:**
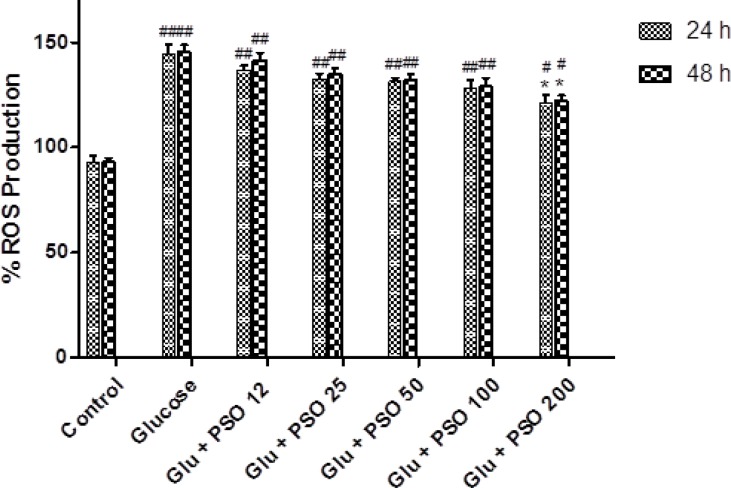
Effect of PSO on glucose-induced ROS generation in H9c2 cells. The cells were pretreated (for 24 and 48 hr) with different concentrations of PSO, before exposure (24 hr) to glucose solution (35 mM). Data are expressed as mean ± SEM of three separate experiments. * p<0.05 as compared to glucose-treated group and # p<0.05 and ## p<0.01 as compared to control group

Glucose caused a significant increase in the level of ROS in H9c2 cells as compared to control (145.5±5.3% and 146.6±6.5 % after 24 and 48 hr, respectively; p<0.01). PSO at the concentration of 200 g/ml decreased intracellular ROS level as compared to control (125.5±6.5% and 126.6±5.5% after 24 and 48 hr, respectively; p<0.05) (Figure 10).

**Effect of PSO on Lipid Peroxidation**

**Figure 11:**
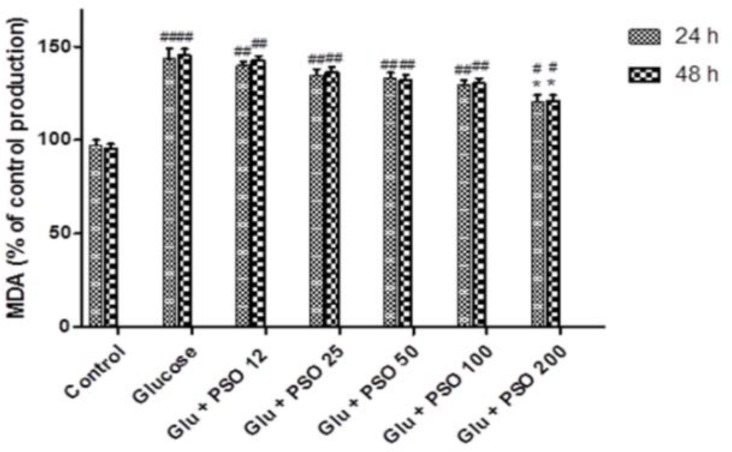
Effect of PSO on glucose-induced MDA production in H9c2 cells. The cells were pretreated (for 24 hr) with different concentrations of PSO before exposure (for 3 hr) to glucose solution (35 mM). Data are expressed as mean ± SEM of three independent experiments. * p<0.05 as compared to glucose-treated group and # p<0.05 and ## p<0.01 as compared to control group

In Figure 11, exposure of the cells to glucose resulted in a significant increase of MDA production (144.7±7.9% and 147.6±6.5% after 24 and 48 hr, respectively, p<0.01) as compared to control. PSO-treated groups showed a significant reduction in MDA level at the dose of 200 g/ml compared to control (122.4±5.5 and 124.5±6.1 after 24 and 48 hr, respectively; p<0.05).

## Discussion

OxS has a major role in the onset and progression of diabetes and its related complications such as retinopathy, neuropathy, nephropathy and cardiovascular diseases (Valko, Leibfritz, Moncol, Cronin, Mazur and Telser 2007 ▶). OxS and ROS can emerge from various diverse sources in diabetic state and tend to the formation of substances that react with cellular proteins, lipids and DNA and disturb their physiological functions. Assessing the activity of antioxidant enzymes in tissues gives a better estimation than measuring them in serum as it depends on many factors including food habits, course of disease, DM complications and laboratory instruments (Rahman 2007 ▶). Because of the fact that mitochondria is a vital cellular organelle that produces energy and ROS and since the heart and kidney are two important organs that suffer from OxS due to DM, this study intended to assess the effects of DM on ROS production and the possible protective role of PSO, as a potent antioxidant agent in this state. 

Safety, low adverse effects, easy to earn and popularity of natural products and their diverse chemical contents have made them interesting for the researchers as an alternative source of curative agents (Afshari, Sadeghnia and Mollazadeh 2016 ▶; Dirin, Mousavi, Afshari, Tabrizian and Ashrafi 2014 ▶). Conjugated linolenic acids (CLnAs) especially punicic acid, are the major compounds in PSO with antioxidant and anti-inflammatory effects. Antioxidant and anti-inflammatory properties of PSO have been mentioned in many reviews. Along these lines, it is reasonable that the utilization of PSO in diabetic condition with excessive OxS status, can ameliorate oxidant/antioxidant imbalance (Boroushaki, Rajabian, Farzadnia, Hoseini, Poorlashkari, Taghavi, Dolati and Bazmandegan 2015 ▶; Schubert, Lansky and Neeman 1999 ▶; Shaban, El-Kersh, El-Rashidy and Habashy 2013 ▶). 

Consequences of this review demonstrated that the changes in oxidant/antioxidant balance and increases in OxS are dependent on the progression of DM. Increased MDA values (as a reliable marker of lipid peroxidation) and decreased total thiol group (a vital part of the structural proteins and non-protein compounds, which might have an essential part in antioxidant enzyme activity) in tissues and mitochondrial fractions are two reasons for proving that the OxS occurs due to the progression of DM ((Mollazadeh, Sadeghnia, Hoseini, Farzadnia and Boroushaki 2016 ▶)). OSI as an OxS marker was increased in diabetic group compared with control group in both tissues and mitochondrial fractions. This expansion was greater after the 4^th^ week as compared to the 3^rd^ week. In spite of the fact that this increase was not statistically significant, this trend was important because of its dependency on the DM progression. Many studies have confirmed that the activity of antioxidant enzymes was reduced in DM (Sheweita, Mashaly, Newairy, Abdou and Eweda 2016 ▶; Tangvarasittichai 2015 ▶; Ullah, Khan and Khan 2015 ▶). The results of this study showed that antioxidant enzymes activity was increased after the 3^rd^ week and it was decreased after the 4^th^ week. This might be due to compensatory upregulation in gene expression and release of the storage form of enzymes but reaction of glucose with enzymes results in destruction of the spatial structure and loss of their activity. The most sensitive enzymes to react with glucose and ROS are SOD, thioredoxine and esterases which finally lost their function (Kang 2003 ▶; Yuan, Jiao, Lau, Wang, Christopher, Lopez, RamachandraRao, Tao and Ma 2010 ▶). 

In the comparable review, lipid peroxidation degree in membrane fatty acids of RBC was related to cell culture glucose concentration and high glucose induced lipid peroxidation (Jain 1989 ▶). Similarly, in STZ- induced diabetic rats, lipid peroxidation degree was associated with plasma glucose level and controlling this, improved the markers of OxS such as MDA (Jain, Levine, Duett and Hollier 1990 ▶).

SOD activity was reduced due to DM. This result was similar to that of other studies (Bray, Cockle, Fielden, Roberts, Rotilio and Calabrese 1974 ▶) but GR, CAT and GPx activity was decreased after the 4^th^ week and if time course of study was longer than 4 weeks, this reduction might become more significant. Kowluru et al. showed that GR, CAT and GPx activity was decreased after 2 months of study in the eye of diabetic rats (Kowluru, Kern and Engerman 1997 ▶). In another study performed on diabetic rats, antioxidant enzymes activity was surveyed weekly in a 6 week period. Results of this study showed that after the first week, this parameter was increased but afterwards, it was decreased weekly to a level lower than that of control group after 6 weeks (Kakkar, Mantha, Radhi, Prasad and Kalra 1997 ▶). SOD and GR are more susceptible to react with ROS and the first defensive enzyme against ROS is SOD. It can be concluded that destruction of SOD by ROS is the main reason of the occurrence of OxS in DM (Aboonabi, Rahmat and Othman 2014 ▶; Bray, Cockle, Fielden, Roberts, Rotilio and Calabrese 1974 ▶). Clinical trials showed that time course of DM and occurrence of its complications are two factors that affect antioxidant enzymes activity. Hence, in the early stages of DM, enzymes activity are risen and after development of complications it will be diminished (Tesauro, Nisticò, Noce, Tarantino, Marrone, Costa, Rovella, Di Cola, Campia and Lauro 2015 ▶).

In the present study, PSO could reduce OSI in both tissues and mitochondrial fractions in a time and dose-dependent manner. This reduction in OSI is especially caused by increases in antioxidant enzymes activity in tissues (TAS) and decreases in ROS production in mitochondrial fraction (TOS). Positive effects of PSO in reduction of OSI are more marked in the heart than kidney. One conceivable reason is the higher number of mitochondria in the heart tissue than kidney due to the higher need for energy in the heart than kidney as well as fatty acid content of mitochondria. Fatty acid content of heart mitochondria is higher than the kidney and this was a predisposing factor for ROS production. 

SOD converts superoxide radicals to hydrogen peroxide which is converted to H_2_O by CAT (Fridovich 1997 ▶). Along these lines, more beneficial outcomes of PSO in ameliorating OxS status have been seen as it enhances SOD and CAT activity. 

Reduced form of GSH is fundamental for cellular antioxidant defense and it is dependent on GR activity. GPx utilizes GSH to reduce H_2_O_2_ and other hydroperoxides to H_2_O (Gill, Anjum, Hasanuzzaman, Gill, Trivedi, Ahmad, Pereira and Tuteja 2013 ▶). In one study, GPx activity and expression, especially GPx1 (an important kidney GPx subunit) have been increased after induction of DM, but after DM progression its activity was diminished (de Haan, Stefanovic, Nikolic-Paterson, Scurr, Croft, Mori, Hertzog, Kola, Atkins and Tesch 2005 ▶). GST augmentation by PSO was a probable mechanism of PSO in decreasing OSI. Diminishing PON1 activity due to DM was shown in many studies and it was in association with the onset of DM complications (Ikeda, Inoue, Suehiro, Arii, Kumon and Hashimoto 2009 ▶; Sampson, Braschi, Willis and Astley 2005 ▶). One conceivable explanation for this state is the influence of DM on HDL content and its structure which is very important for PON1 activity. Consequently, PSO restored PON1 activity by enhancing serum HDL content (Zarei, Fakher, Tabei, Javanbakht, Derakhshanian, Farahbakhsh-Farsi, Sadeghi, Mostafavi and Djalali 2016 ▶).

Results of this study showed that time course of study was important to the development of OxS status. OxS-related markers showed a decreasing trend after the 4^th^ week in comparison with the same data after 3^rd^ weeks. However, these changes were not significant but their decreasing status were important in terms of clinical features in DM. One limitation of our study was the short period of study because of animals' mortality. If it was continued for a period longer than 4 weeks, the results may become more significant in comparison with control and treatment group. 

Our study was the first survey on the effects of PSO on OxS conditions in diabetic state. We did not measure the gene expression of antioxidant enzymes, serum and tissue vitamin E and C values which is considered to be another limitation of our study.

In discussing in vitro studies, description of glucose effect on cell toxicity is very important. Inducing extracellular matrix production and direct toxic effects of glucose on cells are the main cellular toxic effects of glucose. Likewise, glucose induces stress by increasing the expression of NADPH oxidase enzyme complex (an important enzyme in generating ROS) (Khazim, Gorin, Cavaglieri, Abboud and Fanti 2013 ▶; Trachtman, Futterweit and Bienkowski 1993 ▶). In a similar study, toxic cellular doses of PSO (400 and 800 µg/mL) were omitted and the safe doses (12-200 µg/mL) were used in this study (Bihamta, Hosseini, Ghorbani and Boroushaki 2016 ▶). Protective effects of PSO against glucose- induced toxicity were time and dose-dependent. The dose of PSO that inhibited lipid peroxidation and ROS production was 200 µg/ml at which the maximum amount of viability of cells is observed. Thus, it can be concluded that the effect of PSO on H9c2 viability was due to its antioxidant effect and its potency to ameliorate OxS status in cellular medium.

Lack of measurement of cellular SOD and NADPH oxidase activity were two limitations of our *in vitro* study for better understanding PSO protective mechanisms. It is proposed that the effects of PSO on antioxidant enzymes gene expression quantity should be assessed. Also, another study with a period of more than 1 month, evaluation of PSOs effects on transcriptional factors in OxS condition and surveying its effects on other cell lines, could provide better views on mechanism of action of PSO.

The results of this study suggested that PSO has a protective effect against diabetes- induced alterations in oxidant/antioxidant balance and its complications on kidney and heart tissues, mitochondrial fractions and H9c2 cell line.

## Conflict of interest

The authors declare that there is no conflict of interest.

## References

[B1] Aboonabi A, Rahmat A, Othman F (2014). Antioxidant effect of pomegranate against streptozotocin-nicotinamide generated oxidative stress induced diabetic rats. Toxicol Rep.

[B2] Adams LS, Seeram NP, Aggarwal BB, Takada Y, Sand D, Heber D (2006). Pomegranate juice, total pomegranate ellagitannins, and punicalagin suppress inflammatory cell signaling in colon cancer cells. J Agric Food Chem.

[B3] Aebi H (1984). Catalase in vitro. Meth Enzymol.

[B4] Afshari AR, Sadeghnia HR, Mollazadeh H (2016). A Review on Potential Mechanisms of Terminalia chebula in Alzheimer’s Disease. Adv Pharmacol Sci.

[B5] Baynes JW, Thorpe SR (1999). Role of oxidative stress in diabetic complications: a new perspective on an old paradigm. Diabetes.

[B6] Beltowski J, Wójcicka G, Marciniak A (2002). Species-and substrate-specific stimulation of human plasma paraoxonase 1 (PON1) activity by high chloride concentration. Acta Biochim Pol.

[B7] Bihamta M, Hosseini A, Ghorbani A, Boroushaki MT (2017). Protective effect of pomegranate seed oil against H2O2-induced oxidative stress in cardiomyocytes. Avicenna J Phytomed.

[B8] Boroushaki MT, Mollazadeh H, Afshari AR (2016). Pomegranate seed oil: a comprehensive review on its therapeutic effects. Int J Pharm Sci Res.

[B9] Boroushaki MT, Mollazadeh H, Rajabian A, Dolati K, Hoseini A, Paseban M, Farzadnia M (2014). Protective effect of pomegranate seed oil against mercuric chloride-induced nephrotoxicity in rat. Ren Fail.

[B10] Boroushaki MT, Rajabian A, Farzadnia M, Hoseini A, Poorlashkari M, Taghavi A, Dolati K, Bazmandegan G (2015). Protective effect of pomegranate seed oil against cisplatin-induced nephrotoxicity in rat. Ren Fail.

[B11] Bray RC, Cockle SA, Fielden EM, Roberts PB, Rotilio G, Calabrese L (1974). Reduction and inactivation of superoxide dismutase by hydrogen peroxide. Biochem J.

[B12] de Haan JB, Stefanovic N, Nikolic-Paterson D, Scurr LL, Croft KD, Mori TA, Hertzog P, Kola I, Atkins RC, Tesch GH (2005). Kidney expression of glutathione peroxidase-1 is not protective against streptozotocin-induced diabetic nephropathy. Am J Physiol Renal Physiol.

[B13] Dirin MM, Mousavi S, Afshari AR, Tabrizian K, Ashrafi MH (2014). Potential drug-drug interactions in prescriptions dispensed in community and hospital pharmacies in East of Iran. J Res Pharm Pract.

[B14] Forbes JM, Cooper ME (2013). Mechanisms of diabetic complications. Physiol Rev.

[B15] Fridovich I (1997). Superoxide anion radical (O· 2), superoxide dismutases, and related matters. J Biol Chem.

[B16] Gill SS, Anjum NA, Hasanuzzaman M, Gill R, Trivedi DK, Ahmad I, Pereira E, Tuteja N (2013). Glutathione and glutathione reductase: a boon in disguise for plant abiotic stress defense operations. Plant Physiol Biochem.

[B17] Goertz A, Ahmad KA (2015). Biological Activity of Phytochemical Compounds in Pomegranate-A Review. EC Nutri-tion.

[B18] Habig WH, Pabst MJ, Jakoby WB (1974). Glutathione S-transferases the first enzymatic step in mercapturic acid formation. J Biol Chem.

[B19] Ikeda Y, Inoue M, Suehiro T, Arii K, Kumon Y, Hashimoto K (2009). Low human paraoxonase predicts cardiovascular events in Japanese patients with type 2 diabetes. Acta Diabetol.

[B20] Jain SK (1989). Hyperglycemia can cause membrane lipid peroxidation and osmotic fragility in human red blood cells. J Biol Chem.

[B21] Jain SK, Levine SN, Duett J, Hollier B (1990). Elevated lipid peroxidation levels in red blood cells of streptozotocin-treated diabetic rats. Metabolism.

[B22] Johansen JS, Harris AK, Rychly DJ, Ergul A (2005). Oxidative stress and the use of antioxidants in diabetes: linking basic science to clinical practice. Cardiovasc Diabetol.

[B23] Kakkar R, Mantha SV, Radhi J, Prasad K, Kalra J (1997). Antioxidant defense system in diabetic kidney: a time course study. Life sciences.

[B24] Kang JH (2003). Modification and inactivation of human Cu, Zn-superoxide dismutase by methylglyoxal. Mol Cells.

[B25] Khazim K, Gorin Y, Cavaglieri RC, Abboud HE, Fanti P (2013). The antioxidant silybin prevents high glucose-induced oxidative stress and podocyte injury in vitro and in vivo. Am J Physiol Renal Physiol.

[B26] Kowluru RA, Kern TS, Engerman RL (1997). Abnormalities of retinal metabolism in diabetes or experimental galactosemia IV Antioxidant defense system. Free Radic Biol Med.

[B27] Madesh M, Balasubramanian K (1998). Microtiter plate assay for superoxide dismutase using MTT reduction by superoxide. Indian J Biochem Biophys.

[B28] Max SR, Garbus J, Wehman HJ (1972). Simple procedure for rapid isolation of functionally intact mitochondria from human and rat skeletal muscle. Anal Biochem.

[B29] Mollazadeh H, Hosseinzadeh H (2014). The protective effect of Nigella Sativa against liver injury: a review. Iran J Basic Med Sci.

[B30] Mollazadeh H, Sadeghnia HR, Hoseini A, Farzadnia M, Boroushaki MT (2016). Effects of pomegranate seed oil on oxidative stress markers, serum biochemical parameters and pathological findings in kidney and heart of streptozotocin-induced diabetic rats. Ren Fail.

[B31] Moongkarndi P, Kosem N, Kaslungka S, Luanratana O, Pongpan N, Neungton N (2004). Antiproliferation, antioxidation and induction of apoptosis by Garcinia mangostana (mangosteen) on SKBR3 human breast cancer cell line. J Ethnopharmacol.

[B32] Paglia DE, Valentine WN (1967). Studies on the quantitative and qualitative characterization of erythrocyte glutathione peroxidase. J Lab Clin Med.

[B33] Rahman K (2007). Studies on free radicals, antioxidants, and co-factors. Clin Interv Aging.

[B34] Rains JL, Jain SK (2011). Oxidative stress, insulin signaling, and diabetes. Free Radic Biol Med.

[B35] Sampson MJ, Braschi S, Willis G, Astley SB (2005). Paraoxonase-1 (PON-1) genotype and activity and in vivo oxidized plasma low-density lipoprotein in Type II diabetes. Clin Sci.

[B36] Schubert SY, Lansky EP, Neeman I (1999). Antioxidant and eicosanoid enzyme inhibition properties of pomegranate seed oil and fermented juice flavonoids. J Ethnopharmacol.

[B37] Shaban NZ, El-Kersh MA, El-Rashidy FH, Habashy NH (2013). Protective role of Punica granatum (pomegranate) peel and seed oil extracts on diethylnitrosamine and phenobarbital-induced hepatic injury in male rats. Food Chem.

[B38] Sheweita S, Mashaly S, Newairy A, Abdou H, Eweda S (2016). Changes in oxidative stress and antioxidant enzyme activities in streptozotocin-induced Diabetes mellitus in rats: Role of Alhagi Maurorum extracts. Oxid Med Cell Longev.

[B39] TA S (2014). Diagnosis and classification of diabetes mellitus. Diabetes care.

[B40] Tangvarasittichai S (2015). Oxidative stress, insulin resistance, dyslipidemia and type 2 diabetes mellitus. World J Diabetes.

[B41] Tesauro M, Nisticò S, Noce A, Tarantino A, Marrone G, Costa A, Rovella V, Di Cola G, Campia U, Lauro D (2015). The possible role of glutathione-S-transferase activity in diabetic nephropathy. Int J Immunopathol Pharmacol.

[B42] Trachtman H, Futterweit S, Bienkowski RS (1993). Taurine prevents glucose-induced lipid peroxidation and increased collagen production in cultured rat mesangial cells. Biochem Biophys Res Commun.

[B43] Ullah A, Khan A, Khan MI (2015). Diabetes mellitus and oxidative stress―a concise review. Cell.

[B44] Valko M, Leibfritz D, Moncol J, Cronin MT, Mazur M, Telser J (2007). Free radicals and antioxidants in normal physiological functions and human disease. Int J Biochem Cell Biol.

[B45] Vroegrijk IO, van Diepen JA, van den Berg S, Westbroek I, Keizer H, Gambelli L, Hontecillas R, Bassaganya-Riera J, Zondag GC, Romijn JA (2011). Pomegranate seed oil, a rich source of punicic acid, prevents diet-induced obesity and insulin resistance in mice. ‎Food Chem Toxicol.

[B46] Yuan Y, Jiao X, Lau WB, Wang Y, Christopher TA, Lopez BL, RamachandraRao SP, Tao L, Ma X-L (2010). Thioredoxin glycation: A novel posttranslational modification that inhibits its antioxidant and organ protective actions. Free Radic Biol Med.

[B47] Zarei M, Fakher S, Tabei SMB, Javanbakht MH, Derakhshanian H, Farahbakhsh-Farsi P, Sadeghi MR, Mostafavi E, Djalali M (2016). Effects of vitamin A, C and E, or omega-3 fatty acid supplementation on the level of paraoxonase and arylesterase activity in streptozotocin-induced diabetic rats: an investigation of activities in plasma, and heart and liver homogenates. Singapore Med J.

